# Multiplex Detection of Gene Expression in the Intact *Drosophila* Brain Using Expansion-Assisted Iterative Fluorescence *In Situ* Hybridization

**DOI:** 10.3791/67656

**Published:** 2025-05-02

**Authors:** Kari Close, Yisheng He, Jennifer Jeter, Gudrun Ihrke, Mark Eddison

**Affiliations:** 1 Project Technical Resources, Janelia Research Campus, Howard Hughes Medical Institute; 2 Project Pipeline Support’, Janelia Research Campus, Howard Hughes Medical Institute

## Abstract

Understanding gene expression is essential for deciphering cellular functions. However, methods for analyzing the expression of numerous genes *in situ* within a given tissue remain limited. The EASI-FISH protocol described here has been adapted to detect the expression of dozens of genes in the intact adult *Drosophila* central nervous system (CNS) using commercially available reagents. This protocol includes a new gel formulation that enhances gel robustness, enabling multiple rounds of hybridization and allowing the embedding of multiple brains per gel. This improvement increases throughput, facilitates optimal comparison of experimental conditions, and reduces reagent costs. Additionally, by employing the GAL4-UAS system for co-detection of green fluorescent protein (GFP), gene expression can be visualized in specific neuronal or glial cell types. Notably, the high resolution achieved through expansion microscopy, combined with the sensitivity of the method, allows for the detection of single RNA transcripts. This approach effectively integrates high image quality with high throughput, making it a powerful tool for studying gene expression throughout the intact fly brain.

## Introduction

FISH was first utilized in *Drosophila* over 40 years ago to map genes on polytene chromosomes^1,2^. Today, single-molecule FISH is the gold standard for spatially localizing and quantifying mRNA transcripts in intact tissue^3^. However, due to issues of sample integrity and RNA loss, whole mount single molecule FISH techniques in *Drosophila* have been limited to only a few genes that can be analyzed in the same sample^4,5,6,7^. While the recent development of barcoding (e.g.^8,9,10^) increases the number of transcripts that can be detected up to several hundred even in thick tissue^11,12^, the establishment of such protocols requires a considerable investment of resources, which could be a hurdle to implementation. Indeed, many inventive spatial transcriptomic techniques never spread beyond their institutions of origin^13^. Therefore, a straightforward and comparatively inexpensive method to detect the expression of dozens of genes in the same intact brain would facilitate various research objectives: validation of RNA seq and cell atlas data, mapping of molecular cell types across the CNS, quantifying developmental, sex or species-specific differences in gene expression, or measuring changes in gene expression induced by the large variety of genetic or environmental perturbations available in *Drosophila*.

Expansion microscopy is a technique that allows researchers to observe tissues at higher resolution by expanding them in a swellable hydrogel^14^. EASI-FISH combines expansion microscopy and RNA FISH^15^, which utilizes the hybridization chain reaction (HCR), a non-enzymatic technique to detect oligo probe binding^16^. EASI-FISH was first developed as an alternative approach to thin tissue FISH to map expression patterns of dozens of genes in thick (300 μm) tissue slices of mouse lateral hypothalamus^17^. This method is ideal for application to adult fly brains, given this tissue is less than 300 μm thick, and a prototype fly EASI-FISH protocol has recently been described^18^. Here, dissected and fixed *Drosophila* brains are embedded in a swellable hydrogel, where mRNA and proteins are covalently anchored to the gel polymer with the alkylating agents Melphalan-X and Acryloyl-X (Ac-X), respectively. After protein digestion, which allows for isotropic expansion of the brain, gene-specific DNA oligo probes are hybridized to mRNA in the tissue to locate transcripts of interest. Fluorescently tagged DNA hairpins are then added in a second hybridization step. Multiple hairpin molecules polymerize in the HCR reaction to form meta-stable DNA polymers. This leads to an amplification of the signal for individual transcripts, which can be detected as bright spots by light sheet or confocal microscopy.

The covalent anchoring of mRNA and protein to the gel matrix and proteolytic digestion of proteins after gel formation creates an environment where mRNA transcripts are relatively stable and potentially more accessible to probe binding. The stability of mRNA and the gel permits multiple rounds of re-probing and imaging, with the stripping of DNA probes and HCR product by incubation with the enzyme DNase1 between rounds^15,17^. Furthermore, expansion of the sample reduces autofluorescence and light scattering by molecular de-crowding and effectively increases image resolution^14^. Therefore, individual mRNA transcripts can be more easily resolved.

GFP sustains the procedure sufficiently to remain detectable by fluorescence or post-expansion antibody binding. Therefore, this reporter can be used to mark and segment cell types of interest. In conjunction with the many neuron-specific split-GAL4 driver lines available (e.g.,^19,20^), this opens up the possibility of gaining further insight into the molecular identity of a neuronal type or identifying molecular subtypes that cannot be easily distinguished by their anatomy.

Described here is an updated version of the prototype fly EASI-FISH protocol^18^ that features excellent mRNA retention, removal of technical difficulties, and greater robustness of the gel. Modifications include increasing the PFA concentration, lowering the temperature of fixation, and facilitating sample preparation by mounting the brains before incubation with Melphalan-X and Ac-X. This protocol also incorporates a new gel recipe to increase gel robustness, which effectively reduces gel damage when performing many rounds of FISH and imaging. These modifications make the protocol more straightforward to perform, decreasing both processing time and reagent use, and enhancing confidence in consistent results. Importantly, the capability of multiplexing is increased such that dozens of genes can be investigated in the same brain over a period of months. This is illustrated by the expression patterns of several neuronal genes in the adult *Drosophila* brain across a range of expression levels.

## Protocol

**Figure 1:**
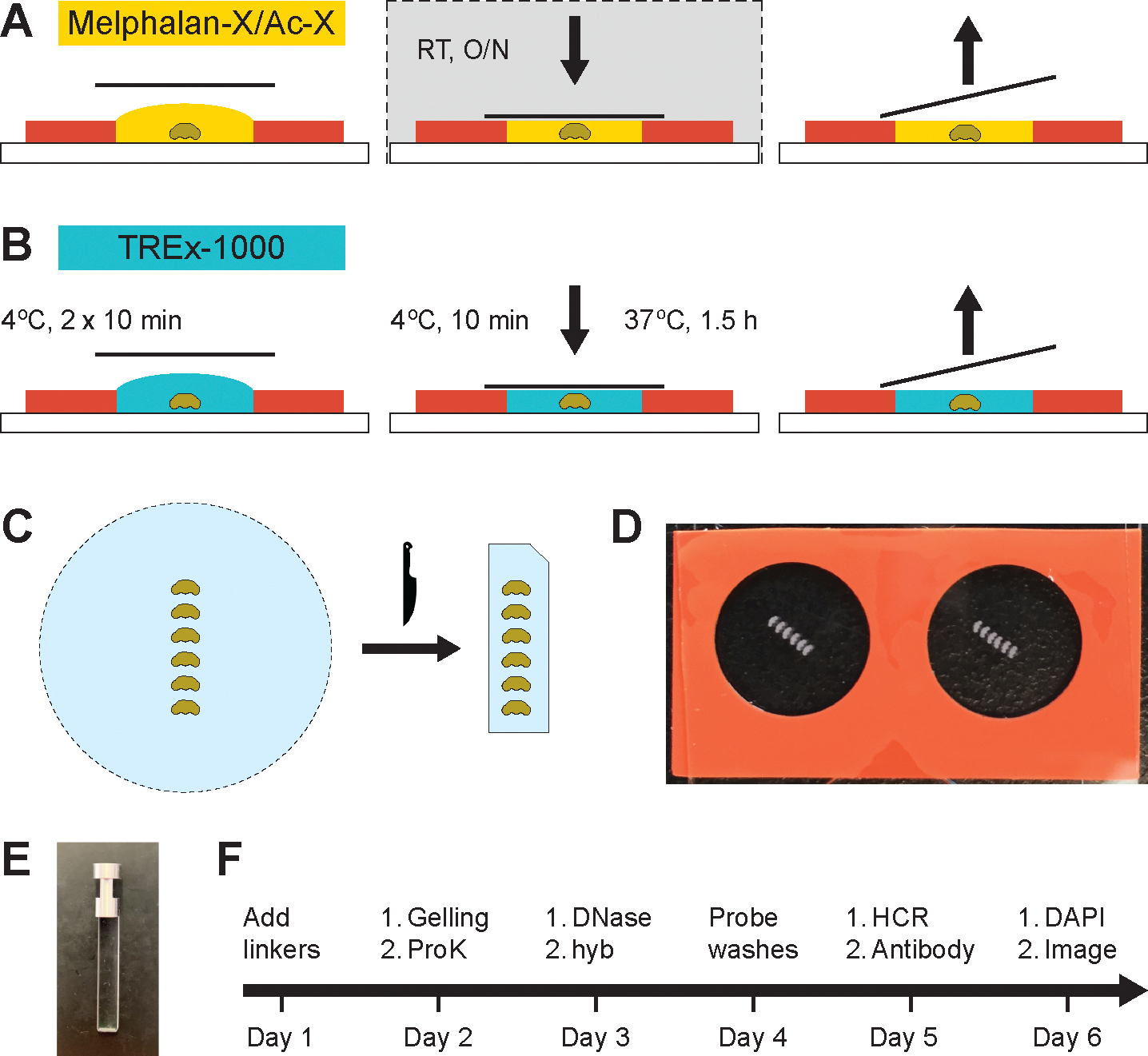
Steps in brain embedding. (**A**) Brains are mounted on a poly-lysine-coated slide in the center of a chamber prepared prior to the experiment. The Melphalan-X/Ac-X solution (RNA and protein linkers) is added, and a coverslip is placed over the chamber to seal without adhesive. The sample is incubated overnight at room temperature in a humidified chamber. The following day, the coverslip is removed, and the Melphalan-X/Ac-X solution is discarded. (**B**) The TREx-1000 gel solution is added and incubated for 10 min (2x) intervals at 4 °C. The gasket adhesive on the top surface is exposed by removing the protective film, a fresh gel solution is added, and the chamber is sealed with a coverslip. The chamber is incubated for a final 10 min at 4 °C before polymerizing the gel at 37 °C for 1.5 h. The coverslip and silicone gasket are removed using a new razor blade to recover the gel. (**C**) The gel is trimmed into a rectangle using a razor blade, and the upper right-hand corner is cut to ensure proper sample orientation during imaging. (**D**) Photograph showing two chambers on a single slide with six mounted brains per chamber. (**E**) Photograph of a gel holder for the light sheet microscope. (**F**) Timeline of the EASI-FISH protocol.

This protocol was performed on adult (5–6 day old) *Drosophila melanogaster* females, but any sex or *Drosophila* species is applicable. For gel anchor recipes, see Supplementary Table 1; for the gel recipe, Supplementary Table 2; and for other EASI-FISH reagents, Supplementary Table 3. For a day-to-day materials and reagents guide, see Supplementary Table 4. For light sheet microscope gel holder, see **Supplementary File 1**. Figure 1A–C shows a schematic depiction of the embedding of brains in gels before expansion and probe hybridization (steps 2.1 through 3.8). Figure 1D is a photograph of the embedded brains, Figure 1E shows a gel holder for light sheet microscopy, and Figure 1F depicts the timeline of a typical experiment. The commercial details of the reagents and the equipment used are listed in the Table of Materials.

### 1. Fly brain dissection and fixation

1. Dissect fly brains or CNS in cold S2 medium (for more dissection details, see reference^21^)
2. To fix the brains, add up to 20 brains or 10 CNS to 2 mL of cold 4% PFA/PBS and incubate overnight on a nutator at 4°C.
  CAUTION: PFA is harmful if swallowed or inhaled, irritant to skin and eyes, and may be carcinogenic.
3. Rinse the sample (1x) with 2 mL of cold PBT (0.5%, see Supplementary Table 4), then wash the sample with 2 mL of cold PBT (4x) for 15 min on a nutator at 4 °C.
4. Rinse the sample (1x) with 2 mL of cold 70% EtOH. Store the brains in 2 mL 70% EtOH at 4 °C for up to 6 months.

### 2. Mounting brains and adding RNA and protein linkers (Day 1)

NOTE: Wear gloves at all times and consistently wipe down equipment and workspace with RNase Away. Gloves are required (1) to keep RNases away from samples and (2) as protection from several reagents that pose a health hazard.

1. Prepare gel chambers by wiping a non-charged slide with an RNase decontamination reagent. Remove the opaque film protecting the adhesive from the silicone gasket, adhere the gasket (with up to 4 chambers) to the slide, and coat each chamber surface with 1 μL poly-lysine using a P20 pipette tip. Air dry and repeat the process.
  NOTE: Step 2.1 can be done the day before.
2. Transfer the brains to a 0.2 mL PCR tube (up to 4 brains per tube) and rehydrate for (2x) 5 min in 150 μL of PBT (0.1%) and (2x) for 5 min in PBS.
3. To mount the brains, add 40 μL of PBS per chamber, then gently stick down the brains in a row in the center of the chamber with fine tweezers, leaving some space between the brains (see Figure 1D). Mounting up to 6 brains or 2 CNS per chamber is possible.
4. Remove PBS and immediately add 50 μL of 20 mM MOPS buffer. Incubate for 30 min at room temperature (RT).
5. While brains are equilibrating in MOPS buffer, thaw the Melphalan-X and Ac-X stock solutions and prepare 75 μL of Melphalan-X/Ac-X solution per chamber (dilute Melphalan-X stock 1:1 with MOPS buffer and add Ac-X at 1:100). Vortex and spin briefly to collect the solution; time the preparation such that the solution is ready at the end of the 30 min incubation time, not earlier.
  CAUTION: Melphalan-X is harmful if swallowed or inhaled, irritant to skin and eyes, and may be carcinogenic.
6. Remove all MOPS buffer and add 50 μL of Melphalan-X/Ac-X solution.
7. Cover the chamber by placing a coverslip on top, but do not use the gasket adhesive to seal. Place the chamber in a humidified box and incubate protected from light overnight at RT.

### 3. Gelation and Proteinase K digestion (Day 2)

1. Carefully remove the coverslip and Melphalan-X/Ac-X, and wash mounted brains (2x) for 2 min with 100 μL of PBT (0.1%) and (1x) for 2 min with 100 μL of PBS.
2. Thaw TREx-1000 solution, 4HT, TEMED, and APS and keep on ice.
  NOTE: TREx-1000 can precipitate at −20 °C; vortex well to completely dissolve after thawing.
  CAUTION: These reagents are harmful or toxic when swallowed or if inhaled, as well as skin and eye irritants.
3. Prepare the gel solution by mixing TREx-1000 with 4HT, TEMED, and APS at a ratio of 94:2:2:2. Vortex and keep on ice. For each chamber, prepare 150 μL of gel solution.
4. Remove PBS from the chamber with a pipette tip and carefully wick away any remaining PBS with a lint-free wipe. For each chamber, immediately add 40 μL of gel solution on top of the brains. Incubate at 4 °C for 10 min.
5. Remove the gel solution and add 40 μL of fresh gel solution, incubating at 4 °C for another 10 min.
  NOTE: Collect gel waste solution in a microcentrifuge tube and polymerize before discarding.
6. Remove the gel solution and peel the clear protective film off the gasket’s surface adhesive. Add a final 45 μL of fresh gel solution, gently place a coverslip over the chamber, and avoid trapping any air bubbles. Press the coverslip to seal the chamber and incubate at 4 °C for 10 min (for a total of 30 min equilibration at 4 °C).
7. Polymerize the gel at 37 °C for 1.5 h.
8. Once the gels have set, allow them to cool on the bench for a few minutes. Carefully take off the coverslip and remove the silicone gasket with a new razor blade. Next, trim the gel into a rectangle and cut the top right-hand corner to track sample orientation.
9. Remove the gels from the slide with a fine paintbrush wetted with a small amount of ProK Buffer and transfer them individually to 2 mL microcentrifuge tubes.
10. Incubate each gel with 500 μL of ProK buffer and 5 μL of ProK enzyme (1:100) at 37 °C overnight.

### 4. DNA digestion and probe hybridization (Day 3)

1. Wash the gels (3x) for 10 min with 1 mL of PBS at RT.
  NOTE: Use fine-tip transfer pipettes to avoid damaging the gel when replacing the solutions.
2. Incubate gels for 30 min in 1 mL of of DNase1 buffer at 37 °C.
3. Add 50 μL of DNase1 enzyme to 450 μL of DNase buffer. Gently mix, but do not vortex. Incubate each gel in 500 μL of DNase1 for 2 h 37 °C. Wash (4x) 15 min with 1 mL of PBS at RT.
  NOTE: Gels can be stored in 5x SSC at 4 °C before or after the DNase digest/washes for several weeks.
4. Thaw hybridization (hyb) and probe wash buffer. Incubate the gels in 500 μL of hyb buffer with no probes for 30 min at 37 °C.
  CAUTION: The hyb and probe wash buffer contain formamide, which is suspected of causing cancer.
5. Dilute the probes 1:100 in hyb buffer (10 nM), preparing 300 μL per gel. Incubate the gels with hyb buffer and probes overnight at 37 °C. Keep the probe wash buffer and PBS at 37 °C.

### 5. Probe washes (Day 4)

1. Wash the gels (3x) for 30 min with 750 μL of probe wash buffer at 37 °C.
2. Wash the gels (3x) for 30 min, then (3x) for 1 h with 1 mL of PBS at 37 °C.
3. Wash the gels in 2 mL of PBS at RT overnight or 4 °C over the weekend.

### 6. Hybridization Chain Reaction (HCR) with fluorescent hairpins (Day 5)

NOTE: For the HCR principle, see reference^22^.

1. Incubate the gels in 500 μL of amplification (amp) buffer for 30 min at RT.
2. Heat the fluorescent hairpins at 95°C for 90 sec in a PCR machine and rapidly cool to 25°C for 30 min.
3. For each probe, dilute matching hairpin pairs (h1 and h2) 1:50 in amp buffer, preparing 300 μL per gel and vortex.
4. Incubate the gels with the hairpin mixture for 3 h at RT in the dark.
5. Wash the gels (2x) for 15 min with 750 μL of 5x SSCT and (2x) for 30 min with 1 mL of 0.5x SSCT at RT.
6. If brains express GFP, stain with 500 μL of anti-GFP-AF488 antibody (1:500) in PBT (0.1%) containing 5 mg/mL of ultrapure BSA and incubate overnight (or over the weekend) at 4 °C.

### 7. Mounting of gels and imaging (Day 6)

1. After antibody incubation, rinse the gels once and wash (1x) for 30 min with 1 mL of PBT (0.1%), followed by (3x) for 30 min with 1 mL of PBS.
  NOTE: Washing in PBS will result in 2x expansion; for 3x expansion, wash with 0.05x SSC.
2. While washing, coat the gel attachment surface of the light sheet microscope gel holder twice with 1 μL of poly-lysine, allowing it to dry at 37 °C between coats. Similarly, for confocal imaging, coat the gel attachment area of a glass bottom dish with repeated applications of poly-lysine.
3. Stain the gels for 30 min at RT in 1 mL of 5 μg/mL DAPI/PBS or DAPI/0.05x SSC, or alternatively, stain overnight at 4 °C.
4. To mount the gel on the gel holder (light sheet microscope) or glass bottom dish (confocal microscope), place the gel on a coverslip and carefully dry the edges with a lint-free wipe. Ensure that the gel is orientated so that the tissue will be closest to the objective. Using a paintbrush, gently place the gel onto the poly-lysine surface in one movement. For imaging, bathe gel in PBS (or 0.05x SSC if expanding to 3x).
  NOTE: After imaging, gels can be gently removed from the poly-lysine surface with a wet paintbrush and transferred to a 2 mL microcentrifuge tube.
5. For immediate re-probing, proceed to day 7. For longer-term storage (1 week to 6 months), store gels in 1 mL of 5x SSC at 4 °C. When ready to use, wash gels (3x) for 10 min with 1 mL of PBS before proceeding.
  NOTE: The extent of expansion is generally consistent in the buffers described (~2x in PBS and ~3x in 0.05x SSC). To check the expansion factor, measure the size of a brain before and after expansion in images taken at a fluorescence stereomicroscope. To visualize the brain after expansion, briefly stain it with DAPI. The approximate expansion factor is the post-expansion size divided by the pre-expansion size.

### 8. Stripping probes and hairpins for multiplexing (Day 7)

1. Incubate the gels for 30 min in 1 mL of DNase1 buffer at 37°C.
2. Add 50 μL of DNase1 to 450 μL of DNase buffer. Gently mix, but do not vortex. Incubate the gels in 500 μL of DNase1 for 2 h at 37 °C.
3. Wash (4x) for 15 min with 1 mL of PBS at RT. Gels are now ready for the next round of hybridization (Day 3, step 6).

## Representative Results

**Figure 2:**
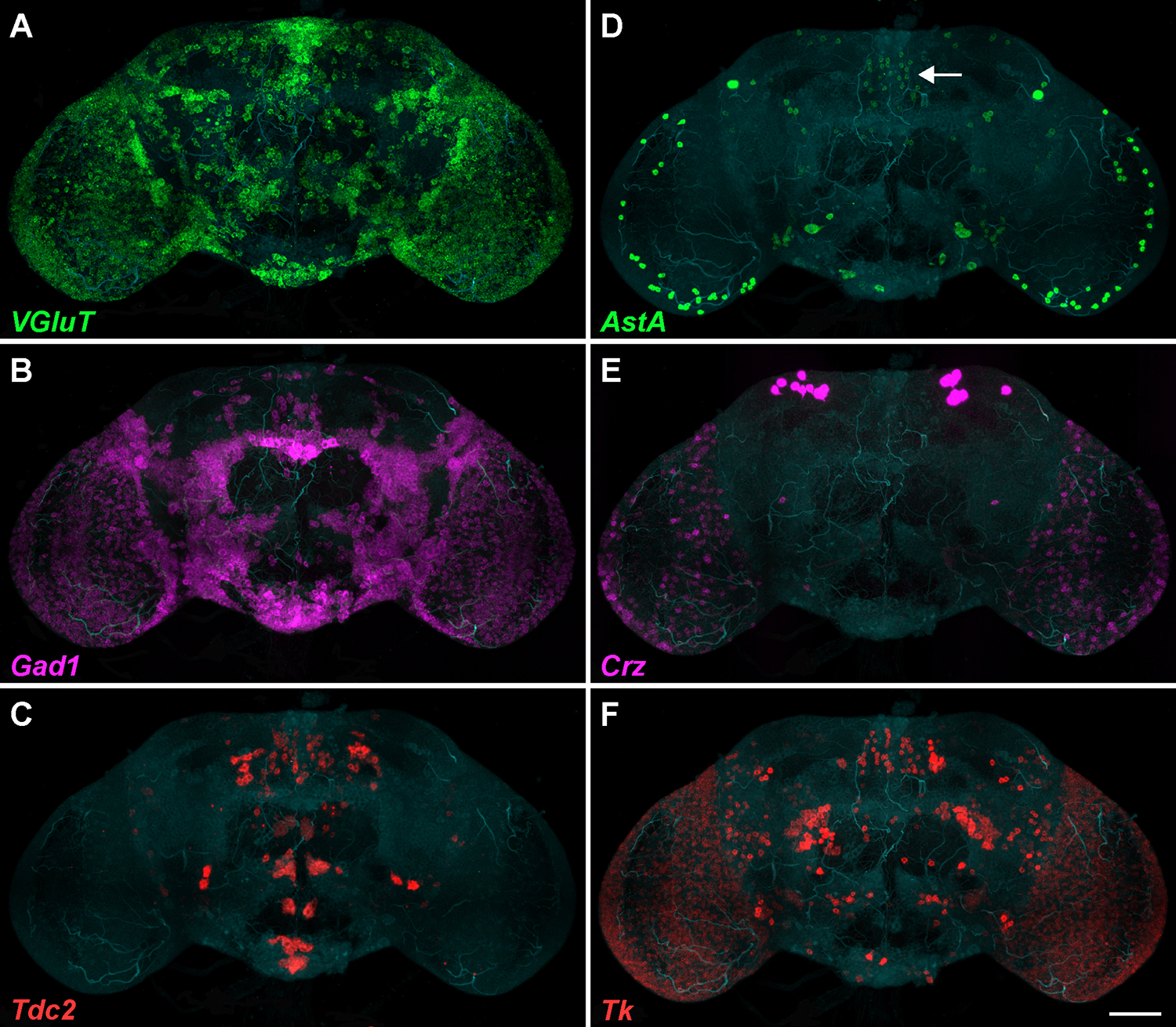
Detection of multiple transcripts in the same *Drosophila* brain using EASI-FISH. Shown are representative examples of maximum intensity projections (MIPs) of multi-tile image acquisitions of the entire adult brain after 2x expansion. The same brain was labeled for three neurotransmitter-associated genes (**A-C**) and three neuropeptide genes (**D-F**), separated by up to six rounds of FISH for other genes (data not shown). Counterstaining with DAPI detects RNA in all cells of the brain; it is presented here in dim cyan to outline the shape of the brain. (**A-C**) Transcripts of (**A**) *vesicular glutamate transporter* (*VGluT)* were detected with AF488, (**B**) *glutamic acid decarboxylase* (*Gad1*) with JF669, and (**C**) *tyrosine decarboxylase 2* (*Tdc2*) with AF546. While *VGluT* and *Gad1* expression patterns are broad (and generally mutually exclusive), *Tdc2* expression is restricted to fewer neurons. **(D-F)** Transcripts of (**D**) *Allatostatin A* (*AstA)*, (**E**) *Corazonin* (*Crz*), and (**F**) *Tachykinin* (*Tk*), detected with AF488, JF669, and AF546, respectively. The expression levels of a given neuropeptide vary over a broad range in different neurons and brain areas. The arrow in (**D**) points to a group of *AstA-*positive neurons in the central complex with much lower expression levels compared to *AstA*-positive neurons in the optic periphery. Note that images were taken below saturation levels (laser power and exposure time were individually optimized), and lower expressing cells were still easily detectable. The brightness levels shown here were chosen so that the varied expressions can be appreciated. Images were acquired sequentially as three separate tracks to avoid bleed-through (track 1, 488 nm and 638 nm lasers; track 2, 561 nm laser; track 3, 405 nm laser) using a light sheet microscope equipped with a 20x water immersion objective. The zoom factor was 1. Images were stitched and processed with Imaris software. The scale bar represents 100 μm in all panels.

**Figure 3:**
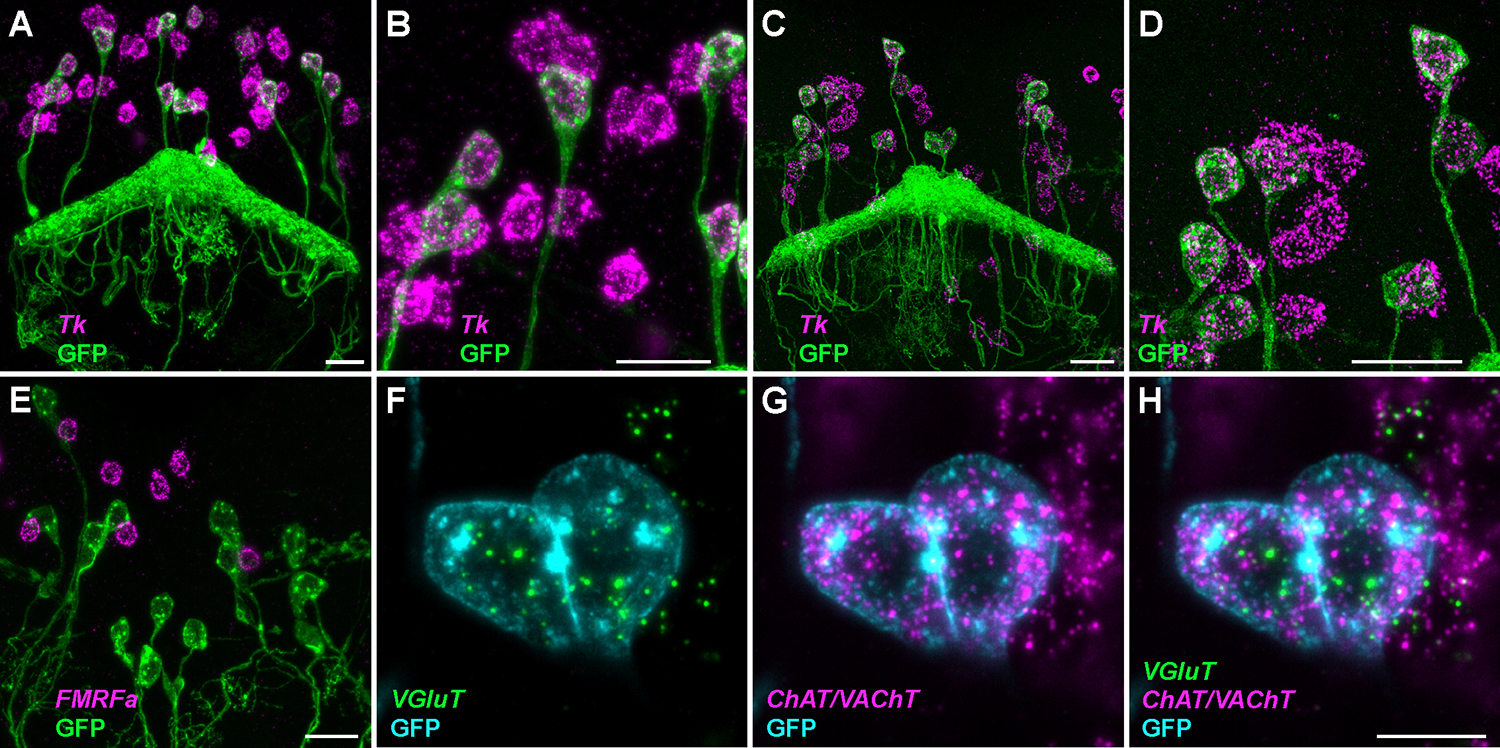
Co-detection of transcripts and GFP to identify neuropeptide and neurotransmitter expression in specific neuron types. Brains were dissected from the progeny of neuron-type specific split-GAL4 drivers crossed to myr-GFP and expanded 2x. MIPs across GFP-expressing cells (**A-E**) or 3.51 μm thin MIPs (in z, composed of nine optical sections) (**F-H**) are shown. **(A-D)** hDeltaD neurons, marked by myr-GFP (green), express *tachykinin* (*Tk*). Images were taken either by light sheet (**A,B**) or confocal microscopy (**C,D**). Five and six of the hDeltaD neurons depicted in (**A**) and (**C**), respectively, are shown at higher magnification in (**B**) and (**D**). Clouds of fluorescent spots, representing single transcripts or clusters, are clearly visible within the cell boundaries of these neurons. Other Tk-positive cells of different neuron type(s) can be distinguished by similar spot clouds, but do not express GFP. (**E**) PFG neurons, marked by GFP, do not express the neuropeptide *FMFRamide* (*FMRFa*). Transcripts can be seen in other cells, but not in GFP-expressing neurons. (**F-H**) FB4K neurons marked with GFP (cyan) express the *vesicular glutamate transporter* [*VGluT* in (F) and (H)], as well as *choline acetyltransferase* and *vesicular acetylcholine transporter* [*ChAT/VAChT*]*,* in (**G**) and (**H**)]. Light sheet imaging was done principally as described in Figure 2 (legend); zoom was 1.5x (**A,B**), 1x (**E**), or 2x (**F-H**). Confocal microscopy (**C,D**) was performed using an inverted microscope equipped with a 40x water dipping objective; the zoom was 0.8x. The images were processed in Fiji^24^. Scale bars represent 20 μm and 10 μm in (**A-E**) and (**F-H**), respectively.

The performance of this modified EASI-FISH protocol is demonstrated by detecting the expression of several different neurotransmitter-associated and neuropeptide genes across the adult *Drosophila* brain over multiple rounds. Typically, gelled and expanded brains were hybridized with two or three probes per round, followed by HCR with hairpin pairs conjugated with Alexa Fluor (AF) 488 (unless GFP was expressed), AF546, and AF647 or Janelia Fluor (JF) 669. Imaging was mostly performed on a light sheet microscope, acquiring a single z-stack or multiple tiles that were stitched together to encompass the entire brain. Single z-stacks were also acquired using confocal microscopy (see details in legends of Figure 2 and Figure 3).

Figure 2 shows examples of neurotransmitter-associated genes (*VGluT, Gad1, Tdc2*) and neuropeptide genes (*AstA, Crz, Tk*) expressed in the *Drosophila* brain. Transcripts were co-detected in the same round of hybridization (*VGluT*/*Gad1* and *AstA*/*Tk*) or in subsequent rounds. Note that six other rounds of FISH and imaging were completed between the detection of the neurotransmitters and neuropeptides shown. All transcripts examined have a distinct distribution across cell types, either broad or more restricted. Areas with no/little fluorescence indicate the absence of transcripts, i.e., the probes were binding specifically to their targets. Importantly, the detection of a wide range of expression levels for a given gene was observed. For example, *AstA* expression is high in some neurons of the optic lobe and relatively low in the central complex (see Figure 2D, arrow points to a group of neurons with lower signal). *Crz* is very highly expressed in some cells in the *pars lateralis*, while numerous cells with lower expression are present in the optic lobes. The completion of up to six rounds of FISH between the examples shown in Figure 2 demonstrates that the possibility of multiplexing is significantly enhanced using the new EASI-FISH protocol. While absolute fluorescent signals may become slightly weaker after many rounds, cells remain generally identifiable as positive.

Split-GAL4 lines were used to drive the expression of plasma membrane-targeted myr-GFP to identify neurons of interest (Figure 3). While endogenous GFP fluorescence is surprisingly well preserved in expansion microscopy, its detection was boosted by incubation with a polyclonal antibody directly conjugated to AF488 after expansion to ensure the best signal-to-noise ratio. Figure 3 illustrates several examples of GFP-expressing neurons at various magnifications. Figure 3A–D shows GFP-expressing hDeltaD neurons in the central complex, which all express *Tk*; several other neurons in the same region (not marked by GFP), also express the gene. Figure 3B and Figure 3D are magnified views of some of the neurons shown in Figure 3A and Figure 3C, respectively. Images in Figure 3C,D were acquired at an inverted confocal microscope, showing principally similar results as those obtained with light sheet microscopy. Figure 3E demonstrates the absence of *FMFRamide* (*FMRFa*) transcripts in PFG neurons marked by myr-GFP, while the prominent signal is seen in the cell bodies of other neurons. At this level of magnification, individual mRNA transcripts are clearly visible as bright puncta in the cell soma around the nucleus (not stained). Figure 3F–H, shows a small substack of images representing a thin slice through two GFP-positive FB4K neurons, where spots can be clearly assigned to individual cells. Results in Figure 3 were generated as part of a larger study of the *Drosophila* central complex^23^.

For transcripts expressed at low to moderate levels, spots are mostly well separated at 2x expansion and countable (note that the images in Figure 3 also show some irregular clusters of spots that are no longer well separated due to brightness enhancement during image processing to show all visible spots). For highly expressed transcripts, such as *AstA* or *Crz* in some neuropeptidergic cells (Figure 2D,E, respectively), individual spots could not be differentiated due to their high density.

## Discussion

The described EASI-FISH protocol for the intact *Drosophila* nervous system enables the detection of dozens of transcripts in the same brain or CNS. It is robust, straightforward, and sensitive, and it can be combined with GFP detection to identify subsets of neurons or glia. Since this method is easy to implement, it should facilitate research by *Drosophila* labs with an interest in mapping and quantifying gene expression in the intact fly brain.

EASI-FISH provides several advantages over previous FISH protocols that do not include expansion microscopy. Excellent RNA stability is observed over many rounds of FISH, as previously reported in mouse tissue^17^. This may be due to the anchoring of mRNAs to the gel matrix, as well as the anchoring and partial digestion of tissue-derived RNases. Similar to other published protocols^5,7,25^, the tissue is fixed here in 4% PFA at 4°C (the prototype protocol used 2% PFA fixation at room temperature for 1 h^18^, a standard method for *Drosophila* immunohistochemistry). The fly brain dissection and fixation can be done well in advance of subsequent steps, as fixed brains can be confidently stored in 70% ethanol. No obvious RNA loss was noticed after six months of storage, as judged by signal strength across different lengths of gel storage. However, no formal measurement of RNA degradation after various storage times was taken. Therefore, caution is warranted if absolute signal strength among conditions is investigated.

While the previous ‘stock-x’ recipe for gels^15^ works for EASI-FISH in *Drosophila*^18^, these gels are prone to damage over multiple rounds, especially when dislodging the gel from the gel holder after imaging. To increase gel robustness, a TREx gel recipe was used (TREx-1000)^26^, which has a higher percentage of monomers (acrylamide and sodium acrylate). The expansion factor of TREx-1000 gels is on par with the stock-x gels (approximately 2x in PBS and 3x in 0.05x SSC), due to a lower concentration of bis-acrylamide cross-linker. Although the TREx-1000 gel can be expanded to ~4x in water, full expansion is not advised because probes and hairpins may start to de-hybridize in the absence of salt. TREx-1000 gels 2x expanded can withstand many rounds of hybridization, washing, and imaging without signs of problematic damage. The gels can be stored in 5x SSC for weeks if there are experimental gaps (before the first round of FISH or between rounds) without obvious detriment to the tissue and transcripts.

This protocol uses commercially available HCR reagents (see Supplementary Table 3), including designed probes, hairpins, and buffers. The reagents are relatively economical (around $50 for two probes, matching hairpins, and all other reagents per gel). Generally, sets of 20 probe pairs per gene work well (we refer to one set as “probe” elsewhere). Transcripts expected to have low expression levels (e.g., neuropeptide receptors) or larger transcripts (>3 Kb) may require larger sets and/or higher probe concentration (16 nM). The hairpins are coupled to AF488, AF546, and AF647. The AF647 hairpins are bright and work well; however, they are prone to photobleaching in the gel^17^ and are, therefore, not ideal for confocal imaging with its slower acquisition times. A good alternative is to use JF669, a more stable far-red fluorophore, which can be conjugated to unlabeled hairpins as previously described^18^. After HCR, gels should be imaged within a week, because hairpins are metastable. Of note is that fragmentation of hairpins can be observed when high laser power is used for imaging^17^. In general, light sheet microscopy is best for the imaging of expanded samples to limit imaging times and photobleaching; however, confocal microscopy may also be used.

For the stitching of multi-tile acquisitions, cytoplasmic DAPI staining^27^ can be utilized using either image analysis software or the mouse EASI-FISH pipeline^17^ (the code is available on GitHub^28^). This pipeline can also be utilized to register images across rounds; however, the additional segmentation module cannot be applied because cells are more tightly packed in *Drosophila* brains. Membrane-bound GFP driven by split-GAL4 can be used to segment cells of interest^29^. Masks can then be created within which to count fluorescent spots for the quantification of transcript expression in cells of interest using methods such as RS-FISH^30,31^. While it is possible to manually create 3D masks, this procedure is too time-consuming to be applied to a large number of cells. Therefore, the development of automated segmentation of cells in the *Drosophila* CNS, followed by spot counting, is currently underway*.* Spot counting is preferred over the measurement of total fluorescence intensity because it is independent of the degree of signal amplification during the HCR reaction. Typically, there is a range of brightness values for the same transcript within the same brain, whereby different cell types may show divergent average values. Nonetheless, total fluorescence measurements may be a valid method for the quantification of expression levels under sufficiently controlled conditions.

The EASI-FISH protocol can potentially be combined with antibody staining following expansion and HCR, as exemplified by anti-GFP labeling. However, it needs to be assessed on a case-by-case basis whether epitopes recognized by an antibody are preserved after digestion with proteinase K. If this is not the case, antibody staining pre-expansion can be attempted; however, to limit mRNA degradation, antibody incubation times should be kept as short as possible, and RNase-free BSA must be used for blocking. In summary, a simple and reliable protocol to assess gene expression of dozens of genes in the intact adult *Drosophila* nervous system is described, and this method is likely transferable to other animals with similar-sized brains.

## Supplementary Material

Supplementary Table 1 **Supplementary Table 1: Linker recipes.** This table lists the chemicals required to anchor RNA and protein to the gel.

Supplementary Table 2 **Supplementary Table 2: Gel recipes.** This table provides the chemical components needed to prepare the TREx-1000 gel.

Supplementary Table 3 **Supplementary Table 3: Other EASI-FISH reagents.** This table includes additional reagents used in the EASI-FISH protocol.

Supplementary Table 4 **Supplementary Table 4: Day-to-day list of materials and reagents.** This table outlines the materials and reagents required for each day of the EASI-FISH protocol.

## Figures and Tables

**Table:** 

Name of Material	Company	Catalogue Number

Paraformaldehyde (PFA)	EMS	15710
S2 Medium	ThermoFisher	21720024
PBS	Fisher	BP24384
MOPs Buffer	Fisher	BP308-100
Acryloyl-X	ThermoFisher	A20770
Melphalan	Caymen Chemicals	16665
Anhydrous DMSO	Invitrogen	D12345
Silicone Gaskets	Invitrogen	P24743
Poly-L-lysine hydrobromide (polylysine)	Sigma	P1524
Photoflo-200	EMS	74257
Acrylic Acid	TCI	A0141
Ammonium persulphate (APS)	Sigma	A3678
N,N,N’,N’-Tetramethylethylenediamine (TEMED)	Sigma	T22500
4-Hydroxy-TEMPO (4HT)	Sigma	176141
40% Acrylamide	Bio-Rad	1610140
N,N Methylene-Bis-acrylamide	Bio-Rad	1610142
5 M NaCl RNase-free	Thermofisher	AM97060G
10X PBS	Fisher	BP 3994
Nuclease Free Water	Ambion	AM9932
Proteinase K	NEB	P8107S
0.5M EDTA, PH 8, RNase free	Invitrogen	AM9260G
UltaPure 10% SDS	Thermofisher	15553027
20X SSC	ThermoFisher	AM 9763
Oligo Probes	Molecular Instruments	https://store.molecularinstruments.com/new-bundle/rna-fish
Hybridization Buffer	Molecular Instruments	https://store.molecularinstruments.com/new-bundle/rna-fish
Probe Wash Buffer	Molecular Instruments	https://store.molecularinstruments.com/new-bundle/rna-fish
Amplification Buffer	Molecular Instruments	https://store.molecularinstruments.com/new-bundle/rna-fish
Unconjugated and Alexa Fluor-conjugated Hairpins	Molecular Instruments	https://store.molecularinstruments.com/new-bundle/rna-fish
ChAT_B1	Molecular Instruments	PRG115
VAChT_B1	Molecular Instruments	RTA904
vGLUT_B2	Molecular Instruments	RTB212
GAD1_B5	Molecular Instruments	RTE581
Tdc2_B5	Molecular Instruments	RTE880
AstA_B2	Molecular Instruments	PRR477
Crz_B5	Molecular Instruments	PRR480
Tk_B5	Molecular Instruments	PRR484
FMRFa_B2	Molecular Instruments	PRR487
JF-669, SE	Tocris	6420
DAPI	Sigma	D9542
GFP Polyclonal Antibody, Alexa Fluor 488	ThermoFisher	A-21311
Ultrapure BSA	ThermoFisher	AM2616
RNase-Free DNase1	Qiagen	79254
0.2 ml PCR tubes	USA Scientific	1402-4700
RNase Away	ThermoFisher	7003
5 ml Transfer Pipet- Fine tip	Global Scientific	134070-S20
10M NaOH	Sigma	72068
Acetonitrile, anhydrous	ThermoFisher	042311-K7
QIAquick Nucleotide Removal Kit	Qiagen	28306
0.5 ml screw-cap microcentrifuge tubes, Amber	USA Scientific	1405-9707
Zeiss LSM 980 confocal Inverted microscope	Zeiss	https://www.janelia.org/node/46947/#lsm980
Zeiss Z7 light sheet microscope	Zeiss	https://www.zeiss.com/microscopy/us/products/light-microscopes/light-sheet-microscopes/lightsheet-7.html
Imarus viewer	Oxford Instruments	https://imaris.oxinst.com/imaris-viewer
Imarus Stitcher	Oxford Instruments	https://imaris.oxinst.com/products/imaris-stitcher
FIJI	Open source	https://imagej.net/software/fiji/
6 Well Glass Bottom Plate	Cellvis	P06-1.5H-N
Zeis Z7 Gel Holder	Janelia	CAD files avaiable on request
